# Creation of Mice Bearing a Partial Duplication of HPRT Gene Marked with a GFP Gene and Detection of Revertant Cells *In Situ* as GFP-Positive Somatic Cells

**DOI:** 10.1371/journal.pone.0136041

**Published:** 2015-08-21

**Authors:** Asao Noda, Hirofumi Suemori, Yuko Hirai, Kanya Hamasaki, Yoshiaki Kodama, Hiroshi Mitani, Reid D. Landes, Nori Nakamura

**Affiliations:** 1 Departments of Genetics, Radiation Effects Research Foundation, 5–2 Hijiyama Park, Minami-ku, Hiroshima, 732–0815, Japan; 2 Laboratory of Embryonic Stem Cell Research, Stem Cell Research Center, Institute for Frontier Medical Sciences, Kyoto University, 53 Kawahara-cho, Shougoin, Sakyo-ku, Kyoto, 606–8507, Japan; 3 Department of Integrated Biosciences, Graduate School of Frontier Sciences, The University of Tokyo, Chiba, 277–8562, Japan; 4 Department of Statistics, Radiation Effects Research Foundation, 5–2 Hijiyama Park, Minami-ku, Hiroshima, 732–0815, Japan; University of Texas Health Science Center at San Antonio, UNITED STATES

## Abstract

It is becoming clear that apparently normal somatic cells accumulate mutations. Such accumulations or propagations of mutant cells are thought to be related to certain diseases such as cancer. To better understand the nature of somatic mutations, we developed a mouse model that enables in vivo detection of rare genetically altered cells via GFP positive cells. The mouse model carries a partial duplication of 3’ portion of X-chromosomal *HPRT* gene and a *GFP* gene at the end of the last exon. In addition, although *HPRT* gene expression was thought ubiquitous, the expression level was found insufficient in vivo to make the revertant cells detectable by GFP positivity. To overcome the problem, we replaced the natural *HPRT*-gene promoter with a CAG promoter. In such animals, termed HPRT-dup-GFP mouse, losing one duplicated segment by crossover between the two sister chromatids or within a single molecule of DNA reactivates gene function, producing hybrid HPRT-GFP proteins which, in turn, cause the revertant cells to be detected as GFP-positive cells in various tissues. Frequencies of green mutant cells were measured using fixed and frozen sections (liver and pancreas), fixed whole mount (small intestine), or by means of flow cytometry (unfixed splenocytes). The results showed that the frequencies varied extensively among individuals as well as among tissues. X-ray exposure (3 Gy) increased the frequency moderately (~2 times) in the liver and small intestine. Further, in two animals out of 278 examined, some solid tissues showed too many GFP-positive cells to score (termed extreme jackpot mutation). Present results illustrated a complex nature of somatic mutations occurring in vivo. While the HPRT-dup-GFP mouse may have a potential for detecting tissue-specific environmental mutagens, large inter-individual variations of mutant cell frequency cause the results unstable and hence have to be reduced. This future challenge will likely involve lowering the background mutation frequency, thus reducing inter-individual variation.

## Introduction

Recent genome sequencing studies indicate that normal somatic cells of an apparently healthy individual accumulate different types of somatic mutations; hence somatic cells of an individual are composed of a more or less mosaic structure of somatic mutations [1–6]. Specifically, it is generally thought that cancers start from single stem cells (or progenitors) as a result of multiple mutations accumulated within a cell [7], and that excessive cell growth precedes clinical onset of the disease. Such disease-associated mutations may occur anytime in life; e.g. substantial evidence exists that indicates initial events of some types of childhood leukemia start while in utero [8]. When mutation occurs sufficiently early in life, a large mosaic patch is anticipated; whereas smaller mosaics are anticipated in late-occurring mutations [1]. Also, for certain hereditary diseases, patients’ cells fail to distribute their chromosomes equally to the daughter cells with elevated frequencies; e.g. cultured cells from skin biopsy of Werner syndrome patients [9] as well as somatic cells of patients suffering from variegated mosaic aneuploidy [10] are often mosaic aneuploidy. On the other hand, however, detection of somatic mutations requires careful designs because, for example, a forward mutation causes loss of a gene product in a cell, which may be only detectable when the mutant cells formed a cluster (e.g. loss of color pigment in the hair or skin) and would not be suited at a single cell level because distinctions of the mutant cells from dead or dying cells might not be straightforward. In this regard, reversion mutations (restoration of a gene function) are interesting because normal cells are defective for the gene function in concern whereas mutated cells are positive to the gene products. Generally, reversion mutations occur much less frequently than forward mutations because any inactivating mutations may contribute to forward mutations but reversion mechanisms are seriously restricted depending on the nature of the mutated allele (obviously deletion mutations are the most difficult to restore its normal function). There is one exception though; namely, a mutated loss-of-function allele caused by a partial duplication of the gene. Such alleles are historically well known from bacteria to fruit flies, and they revert to the normal allele by losing one extra copy of the duplication at an elevated frequency, occasionally comparable to or even higher than that of forward mutations. In the laboratory mouse, several mutation assay systems are reported that include both forward and reversion mutations to observe somatic mutant cells in situ, but observations are generally restricted to certain tissues (see below).

Pink-eyed unstable (*p* ^un^) is one of the alleles at the *p* locus which encodes a protein responsible for assembly of a black color melanin complex. The *p* ^*un*^ allele consists of a tandem duplication of 70 kb segment within a gene; characteristically, the allele reverts to wild type with unusually high frequencies [11–13]. In vivo detection of cells that had undergone reversions at the *p* ^*un*^ allele is possible in retinal cells and hair follicle cells. Even though detection of single mutants is possible in retinal cells, studying hair mutations is more difficult since single hair follicles contain multiple stem cells (an exception is mutation induction at an early stage of embryo/fetal life when the stem cells number is still small). Another system that detects forward mutations is the *Dlb-1* gene which specifies reactivity to a lectin; namely, *a* allele product does not react to the lectin while *b* allele product does. Thus, loss-of-function mutations on the *Dlb-1b* allele can be detected in *Dlb-1a*/*Dlb-1b* heterozygous mice [14–16]. This system can only be used to detect mutations in stem cells of the small intestine as unstained streaks in villi or partially or totally unstained crypts. In both systems, however, applications to a wide-range of internal organs are not possible. The purpose of the present study is to develop a mouse model system which enables in vivo detection of mutant cells in various tissues. In this regard, forward mutation is ideal to use but thus far it has not been successful. Thus, as the first step toward the goal of detecting different types of mutations in vivo, we designed a system to detect reversion mutations that may occur as a result of loss of one extra copy from partially duplicated, loss-of-function allele, and show up as GFP-positive cells.

Use of *GFP* or enhanced yellow fluorescent protein (*EYFP*) gene for detection of mutant somatic cells in vivo is not new. Three systems are reported; i.e. they introduced a construct consisting of a tandem array of two copies of *GFP* or *EYFP* gene, and each copy carries a different mutation [17–20]. In the EYFP mouse, the reporter gene was introduced by DNA injection into fertilized eggs [19]. Hence expression levels of the reporter gene in thus created mice were not assured in all tissues, and can be too low to use in some tissues. The reversions were found to be caused primarily by gene conversions. The problem of tissue restriction was subsequently overcome by introducing the construct into the *ROSA26* locus by gene targeting (RaDR-GFP mouse) [20]. In this system, eleven different tissues were studied and the frequency of revertant cells varied extensively among the tissues as well as among mice. It appears that in the RaDR-GFP system, homologous recombination (including gene conversion) is the major cause of the reversion [20]. In another system, the construct was introduced into *Pim1* locus (DR-GFP mouse) by gene targeting. In vivo detection of revertant cells was not described with this system but primary cultures were used to test for recombination activities [18].

In the present study, we directed our attention to a ubiquitously expressed housekeeping gene, the hypoxanthine-guanine phosphoribosyltransferase (*HPRT*) gene [21], and created a mouse system in which *HPRT* gene was inactivated by a partial duplication spanning from intron 5 to exon 9 (about 8 kb); specifically, first exon 8 was truncated and the neo cassette was conjugated while the *GFP* gene was inserted in frame at the 3’ end of the downstream last exon 9 whose stop codon was eliminated in an aim at producing HPRT-GFP fusion proteins (HPRT-dup-GFP mouse). Compared with previously reported systems, the present reversion system only requires loss of one extra copy of the partially duplicated *HPRT* gene by unequal sister chromatid exchanges or intra-strand recombinations of DNA. For genetically manipulated ES cells, the revertants were all confirmed to have undergone a deletion of one of the two duplicated segments. One unfortunate characteristic of the system was that the endogenous *HPRT* gene function was not strong enough in various tissues in vivo to make the revertant cells fluoresce. Thus, a second gene targeting in the ES cells was needed to increase the expression levels by replacing the endogenous promoter of the *HPRT* gene with a CAG promoter, thus creating the knocked-in mice.

## Materials and Methods

### Mice

**Fig 1:**
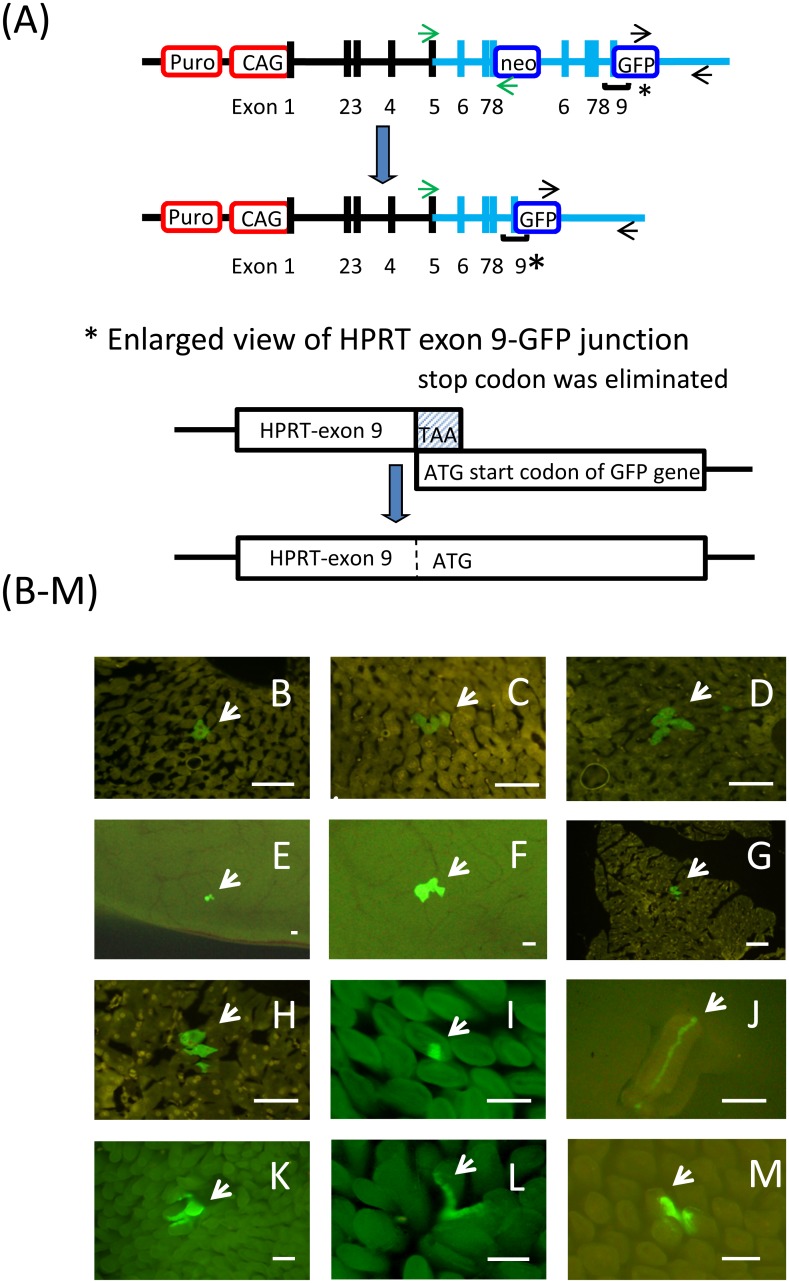
Outline of the HPRT-dup-GFP system and in vivo detection of revertant cells. **A)** Genetic composition of theHPRT-dup-GFP system, **B-D**) Frozen sections of liver, **E-H**) frozen sections of pancreas. Note that nuclei of background GFP-negative cells exhibit yellowish color derived from auto-fluorescence. **F**) and **H**) are enlarged views of **E**) and **G**), respectively (scale bar = 100 μm). **I-M)** whole mount of small intestine; specifically, **J** represent an isolated villus with single mutant streaks (bar = 100 μm), **K)** an example of a whole villus composed of green mutant cells, **L) and M**) are villi of small intestinal with mutant streaks in two adjacent villi (bar = 100 μm). (Note that E and F are squashed samples of pancreas. In these, network of capillary blood vessels is visible.) Arrows indicate mutant(s).

Radiation Effects Research Foundation (RERF) Animal Care and Use Committee approved this research under RERF Research Protocols RP1-08. The outline of creating genetically modified mice is shown in S1 Fig (methods are fully described in S1 File). In brief, a targeting vector was constructed which consisted of a tandem duplication of genomic DNA spanning from intron 5 to exon 8 (7.8 kb) and intron 5 to exon 9 (8.6 kb) of the mouse *HPRT* gene. Floxed Neo marker was also inserted at the 3’ end of the first duplication (at the truncated end of exon 8). Finally, *GFP* ORF was inserted at the 3’ end of the second duplication (exon 9) in frame [(exons 6,7,8-Neo-exons 6,7,8,9-GFP) vector: (S1 and S2 Figs)]. The vector was introduced into ES cells to obtain gene-targeted cells resistant to both Neo (G418) and 6-thioguanine (6TG) (HPRT-dup-GFP cells). The partially duplicated structure of the HPRT gene prohibits normal splicing at the 3’ end of the first exon 8 due to the inserted Neo cassette and multiple stop codons which appear subsequently; thereby the normal mRNA structure is not produced. Thus, structural alterations must be involved in revertant GFP-positive cells. Indeed, all revertant ES cells had lost one copy of the duplicated segments (S3–S6 Figs). A second gene targeting was conducted on the HPRT-dup-GFP ES cells to replace the endogenous promoter of *HPRT* gene with a FRT-puro CAG promoter [22] to obtain CAG-HPRT-dup-GFP ES cells (Fig 1 and S2 Fig). Subsequently mice were obtained following injection of the ES cells to blastocysts and breeding of chimeric F1 animals.

#### Mutant mice bearing knockout alleles of p53 or ATM gene

ATM heterozygous mice (C57BL/6J) or p53 heterozygotes (either C57BL/6J or ICR background) were obtained from RIKEN Institute (Tsukuba and Kobe), and were used to produce offspring bearing both the CAG-HPRT-dup-GFP allele and ATM or p53 knockout allele(s).

### X irradiation

Individual mice of about 8 weeks old were put into a sterile plastic box and were subjected to X-irradiation (220 kVp, 8 mA with a 0.5 mm Al and 0.3 mm Cu filter) using a Shimadzu-Pantak generator (Kyoto, Japan) at a dose rate of 1 Gy/min without anesthesia. A part of the study used another X-ray generator (Faxitron CP-160, Tucson, Arizona) operated at 160 kVp and 6.3 mA with a 0.5 mm Al and 0.21 mm Cu filter at a dose rate of 1 Gy/min. Mice were sacrificed to measure mutation frequencies 3 to 5 months after the irradiation.

### Detection of green mutant cells in vivo

#### Liver and pancreas

Tissues were fixed with 4% paraformaldehyde/PBS for 1 to 2 days at room temperature, and frozen at −80°C until use. Serial 60 cryostat sections (5 μm thickness) were screened under a fluorescent microscope (Nikon Eclipse E600, and later Olympus VS120 Virtual Slide system) with a magnification of 200 to 400×. One section of liver of approximately 1 cm × 1 cm was estimated to contain roughly 1.5×10^5^ cells; hence, 60 sections contained about 9×10^6^ cells. However, as the sections were serial, one cell could appear in three successive sections. The number of cells screened per liver sample was then regarded as 3×10^6^ cells–one third of 9×10^6^. In the pancreas, cross section areas were nearly half the size of the liver’s; thus 1.5×10^6^ cells were regarded as screened per sample. In both liver and pancreas, revertant cells were observed either as solitary green cells or as clusters of green cells. When successive slices contained revertant cells at similar locations of the tissue, we assumed the mutation occurred in a stem cell followed by its clonal expansion, and counted such mutations as single events. The mutation frequency is expressed as the number of mutational *events* per 10^6^ cells. Determination of mutant cell locations was easy because liver and pancreas showed background pattern as seen in Fig 1B–1D,1G and 1H thanks to the different levels of auto-fluorescence among different types of normal cells. Likewise, when multiple clusters of green cells were observed distantly apart within a section, they were counted as independent events. On the other hand, when only a few green cells were seen per slice at a location and were not touching each other, they were regarded as single events followed by slight movements of daughter cells after a few rounds of cell division.

#### Small intestine

Tissue lumens were washed with PBS and briefly fixed with 4% paraformaldehyde/PBS. Then, about half of the small intestine was excised, opened with scissors, fixed with 4% paraformaldehyde/PBS for 1–2 days, and screened for green streaks among the villi using a dissecting fluorescent microscope (Olympus SZX10). The mutant cell frequency was estimated by dividing the count of green streaks (counting streaks derived from a single crypt as one event) by the number of stem cells in crypts. In the literature, murine small intestine contains about 1.1 million crypts and each crypt is supported by 4 stem cells (range 4 to 16) [23]. While another report indicated 10 stem cells per crypt [24], we adopted 4 cells per crypt and estimated that about 4.4 million cells per mouse small intestine. As we observed half of the intestine (i.e. about 10 cm), we assumed 2.2 million crypt stem cells were screened per sample. Related to the cell number, we note that the stem cell number may not be a fixed value but actually increases following physical or chemical insults to the small intestine [25].

#### Spleen

A flow cytometric method was used to gate single cells by forward and side scatters (FACSCalibur, BD), and the frequency of GFP-positive cells among non-fluorescent splenocytes was estimated among 1.5 million cells analyzed in each mouse.

### Statistical methods

Within each tissue, mutation frequency means were compared between 0 Gy and 3 Gy groups with a Wilcoxon-Mann-Whitney test (a rank-based, nonparametric test for a difference in means between two independent groups). The fold-increase in mutation frequency as a consequence of 3 Gy-exposure to radiation was estimated with the ratio of the 3 Gy mean to the 0 Gy mean; 95% confidence intervals (CIs) for fold-increase were computed using basic bootstrap confidence limits; 10,000 bootstrap samples were taken [26]. All analyses were conducted in SAS v9.2.

## Results

### Creation of double knock-in mice

Revertants from the first knock-in ES cells (HPRT-dup-GFP cells) showed green fluorescence with a mean intensity of about 10 fold higher than that of the parental non-fluorescent cells (results not shown). Such revertant ES cells were HPRT-proficient, and hence sensitive to the toxic drug 6TG. However, no green cells were seen in somatic tissues of mice derived from the ES cells. We assumed that, although the natural promoter of *HPRT* gene is sufficiently strong to let the ES cells be green in vitro, it was not strong enough in vivo because most somatic cells are in a quiescent state and/or background noise (auto-fluorescence) of living tissue may not be negligibly low. To increase endogenous promoter activity in the HPRT-dup-GFP ES cells, a second knock-in process was performed replacing the natural promoter of the HPRT gene with a CAG promoter (a chicken beta-actin promoter and cytomegalovirus enhancer; **22**) (Fig 1A); also see S1 and S2 Figs. Revertants from the thus created ES cells (CAG-HPRT-dup-GFP cells) showed much brighter fluorescent intensities than those from the parental ES cells; e.g., more than a 200-fold increase in the fluorescent intensity (S5 Fig). Following an ordinary injection of the ES cells into blastocysts and breeding chimeric offspring, CAG-HPRT-dup-GFP mice (abbreviated as HPRT-dup-GFP mice) were obtained. With these mice, we could detect GFP-positive revertant cells that had lost one copy of the duplication in all tissues examined except for the brain (Fig 1). Frequencies of green mutant cells were measured using fixed and frozen sections (liver and pancreas), fixed whole mount (small intestine), or by means of flow cytometry (unfixed splenocytes). DNA structures of knocked-in and revertant alleles were confirmed by Southern blot analysis and genomic PCR (S3 and S4 Figs). In the present system, all revertants from the ES cells had lost the neo maker located at the first exon 8 and had expressed GFP gene located at the end of the second duplication (Fig 1), thus indicating that they arose as a result of unequal sister-chromatid exchange or intra-strand crossover of DNA but not by gene conversion. Indeed, HPRT-GFP full length messages (1.7 kb) were present in the revertant ES cells (S6B Fig). Further, total RNA extracted from the tip of a tail (as well as tiny pieces from pancreas, liver or spleen) of the mouse showed an expected size of HPRT-GFP fusion mRNA by real-time RT-PCR (S6A and S6B Fig). Also, the data from quantitative real-time RT-PCR indicated that the tail tip contained revertant cells expressing the HPRT-GFP mRNA at a frequency of 10^−5^ to 10^−6^ when compared with the mRNA isolated from revertant ES cells (S6C Fig). Possible mechanisms to give rise to HPRT-GFP fusion proteins are illustrated in S7A and S7B Fig.

### Detection of mutational events or mutant cells

**Fig 2:**
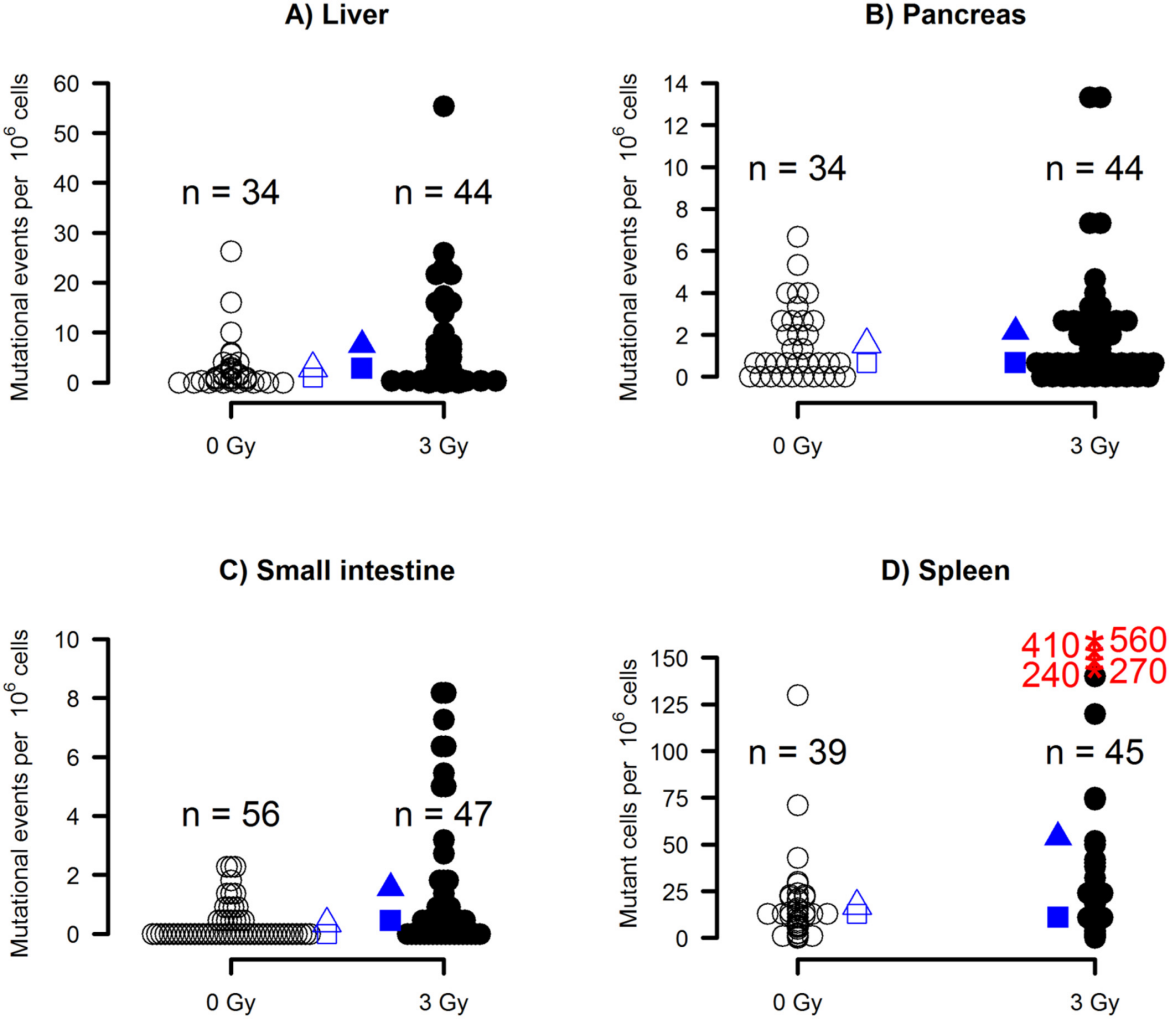
Distributions of mutational events per 10^6^ cells examined. In the liver **(A)** and pancreas **(B)**, frozen sections were scored under a fluorescent microscopy and a cluster of GFP-positive cells that were interrelated across slices were counted as a single event. In small intestine **(C)**, green streaks that appeared in villi were scored using a dissecting microscopy. The results were used to estimate mutational events per 10^6^ crypt stem cells (see Materials and Methods). In spleen (**D**), mutant cells were detected by a flow cytometer and the frequency was expressed per 10^6^ cells. Open and filled circles represent animals receiving 0-Gy and 3-Gy irradiation, respectively. Triangles and squares represent means and medians, respectively. Asterisks (panel D) represent extreme outliers, with their actual values provided in the figure. No extreme jackpot mutations were detected in this series of the experiments.

As mentioned earlier, in liver and pancreas GFP-positive cells that appeared at similar locations in successive sections were considered as clonal descendants and were counted as single events. In contrast, for the small intestine, mutational events were scored as green streaks in the villi and the frequency was estimated per 10^6^ crypt stem cells; and for spleen cells, frequencies of mutant cells were estimated. The results varied widely among individuals as shown in Fig 2. The mean and median frequencies of each tissue are also shown: frequencies were highest in spleen, followed by liver and pancreas, and lowest in the intestine (Table 1).

**Table 1:** Mean mutation frequency in 10^−6^ compared between control (0 Gy) and irradiated (3 Gy) mice. Means were compared with a Wilcoxon-Mann-Whitney test. Fold increase (irradiated vs. control), along with bootstrapped 95% CIs, are also presented; CIs not containing 1 indicate statistical significance at α = 0.05.

Organ	Irradiated (3 Gy)	Control (0 Gy)	*p*-value*	Fold increase (3Gy/0Gy)	95% CI for fold increase	Medians (3 Gy/0 Gy)
Intestine	1.59	0.34	0.001	4.64	(2.36, 9.35)	0.45 / 0
Liver	7.06	2.83	0.02	2.49	(1.25, 5.63)	2.67 / 1
Pancreas	2.14	1.55	0.66	1.38	(0.78, 2.44)	0.67 / 0.67
Spleen	54.95	16.87	0.27	3.26	(1.45, 6.35)	13 / 11

*Wilcoxon *z*-values: intestine, 3.35; liver, 2.43; pancreas, 0.45; spleen, 1.11.

#### Liver and pancreas

Distributions of mutation frequencies are shown in Fig 2. In liver, both single mutant cells and clusters of mutant cells were observed (Fig 1B–1D) and clusters were counted as single event if they were interrelated across slices (in an extreme case, a cluster extended up to 60 successive slices). In pancreas, the majority of mutants were observed as smaller clusters or as single cells (Fig 1E–1H).

#### Small intestine

In the small intestine, green streaks were scored among villi (Fig 1I–1M). Fig 1J shows a physically isolated villus that contains a single streak of GFP-positive cells, and Fig 1K shows a rare villus that was totally occupied by the revertants. Regardless of the thickness or number of streaks, the count of mutation events increased by one as long as the mutant streaks seemed to start from a single crypt. More often than not, mutant crypts supplied their progeny to two villi instead one, which aligns with published results from other laboratories [23, 27].

#### Spleen

Because mice were examined 3 to 5 months after irradiation and clonal descendants were not distinguishable in the present assay, it is possible that clonal propagation of mutant cells could have occurred. Therefore, individual variations in the mutant cell frequencies include not only induced frequencies but also differences in the clonal proliferation during the post-irradiation period. We also detected GFP-positive cells in thyroid glands, colon, lung, and male germ cells (S8 Fig) but their frequencies were not measured.

### Effect of irradiation

Within each organ, exposure to 3 Gy of radiation was hypothesized to increase frequencies of mutational events or mutant cells over background frequencies and statistical significance was tested (see Fig 2 for the distribution of the frequencies). In liver, clusters of GFP-positive cells were often observed across slices while the number of mutational events could become artificially large if the interrelation between revertants clones were undetected. To avoid any undue influence of extreme counts, rank-based nonparametric statistical methods were used to compare between the control and irradiation groups. Although the increase caused by irradiation did not exceed 5× that of the control animals, it was significant for the liver and intestine, but not for the pancreas. Results for the spleen, however, were inconclusive, with no difference when using Wilcoxon’s test, but statistically different means when using a nonparametric bootstrap (see Table 1).

The range of the mutation frequencies differed both within organs (a radiation-attributable difference) and among organs (an organ-attributable difference). The observed radiation effects also differed among organs, with the lowest fold-increase of 1.38 (pancreas) and a highest of 4.64 (intestine) (Table 1). In small intestine of control mice, we observed on average two villi which bore mutant green streaks originating from single common crypts; specifically, out of 104 mutation events (104 crypts bearing mutant stem cells) originating from 56 control mice, 32% crypts provided mutant cell streaks with single villi, 58% with two villi, and 10% with three or more villi. Exposure to 3 Gy was expected to increase singles as irradiation can hit only one stem cell in a crypt at a time. The observed results from 147 mutational events originating from 47 irradiated mice were in the direction of the expectation: 43% singles, 44% doubles, and 13% triples or more, but the difference was only marginally significant when the results were dichotomized into {1, 2+}, (chi-sq [1] = 2.52, p = 0.11).

### Extreme jackpot mutations

In a few animals, some of the tissues showed too many GFP-positive cells to score. Such events were termed as extreme jackpot mutations and the frequency of mutant cells is roughly 1,000×10^−6^ or higher. They were found among 175 mice in a small-scale pilot study aimed at finding factors that may contribute to the large inter-individual variations in mutation frequencies. We considered factors that may operate from early stages of development; namely, alterations of genetic background (specifically, introduction of knockout alleles of p53 or ATM gene) and irradiation of sires’ spermatogonia (i.e., immature stage in male germ cells). While the results did not indicate any clear effect by deficiency of p53 or ATM functions, or by paternal exposure to radiation (results not shown), we encountered a total 7 mice in which at least one tissue showed the extreme jackpot mutation patterns (S1 Table). Frequencies of mutant cells in different tissues of mice having at least one tissue with extreme jackpot mutations are presented in Table 2.

**Table 2:** Frequencies of mutations in different tissues of mice with extreme jackpot mutations.

Mouse ID *	Frequency of mutational events or mutant cells (per 10^6^ cells)			
	Pancreas	Liver	Intestine	Spleen
#445	1	7	0	>2,000
#413	22	17	>2,000	23
#144	2	>2,000	>2,000	>2,000
#484	1	27	0	~1,600
#22	Not studied	Not studied	0	~1,200
#26	Not studied	Not studied	0	>2,000
#47	1	12	0	1,200

*See S1 Table for more individual information of the mouse.

**Fig 3:**
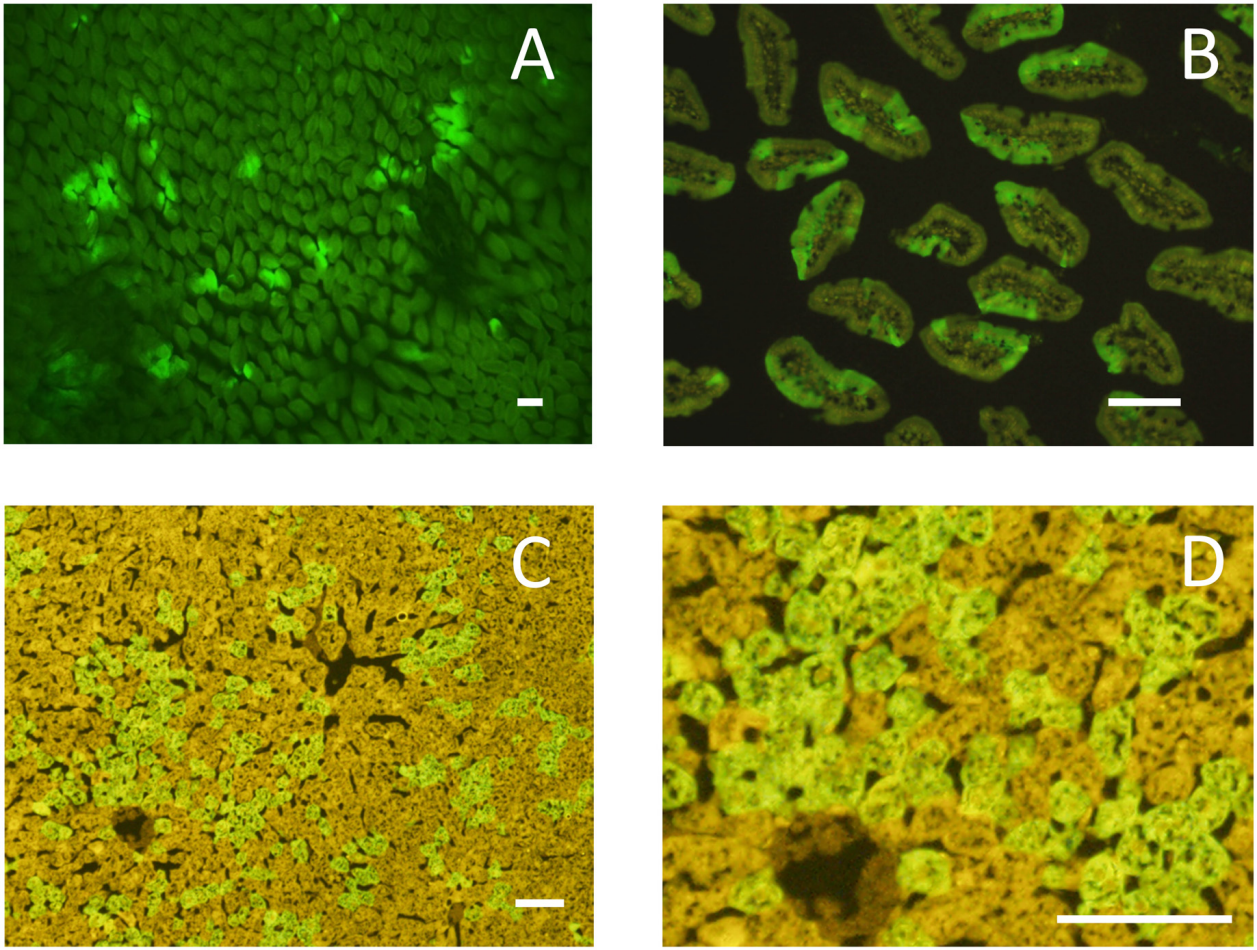
Tissues with extreme jackpot mutations. **(A)** A low magnification view of small intestine under a dissecting fluorescent microscopy. Many mutant green villi are seen. **(B)** A cross section of intestinal villi. Note that mutant GFP-positive cell clusters (bright green sectors) are present in most of the villi. **(C)** A cross section of liver. (**D**) An enlarged picture of (**C**), bar = 100 μm in both (**C)** and (**D**).

It is stressed here that 5 out of the 7 mice showed the extreme jackpot phenotype exclusively in the spleen. Only two mice had this type of mutation in tissues other than spleen; namely, one involved the liver, small intestine, and spleen, and the other involved only the small intestine (Fig 3A and 3B). If we take into account only those extreme jackpot mutations that occurred in tissues other than the spleen, the overall frequency is 2 mice in 278 or roughly 1% (S1 Table, last line). Because the number of events observed is so small, causal relationships between the observations and specific treatments (i.e. altered genetic background or paternal exposure to radiation) are not clear. As this event is most likely to be associated with developmental processes, it is unlikely that adult exposures to ionizing radiation can induce the phenotype. Further, it is also noted that no large mass of green mutant cells was observed in the liver or small intestine of these animals, which indicates that descendants of a mutant stem cell are not restricted to locate side by side but may move slightly so that the tissue shows intermingled mosaic structures.

## Discussion

### Comparisons with previous studies

In the present study, we showed characteristics of new knock-in mice bearing a partial duplication of about 8 kb at the 3’ portion of the endogenous *HPRT* gene (HPRT-dup-GFP mouse). As the *GFP* gene was inserted in frame at the end of the duplication, loss of one copy of the duplicated segments (reversion) resulted in the expression of HPRT-GFP fusion proteins, which made the cells GFP positive. Such GFP-positive cells were detected in various tissues (with the brain an exception) while the frequency varied widely not only among tissues of the same animals but also among the same tissues of different animals. The results are in agreement with those obtained from studies of pancreas in EYFP or RaDR-GFP mice [17],[19],[20] except that the nature of the revertants were different; namely, the revertants arose mainly via gene conversions in the EYFP system while intra-strand exchange or sister-chromatid exchange also contribute in the RaDR-GFP system. In either system including ours, detecting mutagenic action of radiation was hampered by large variations of mutant cell frequencies among animals. Compared with the results obtained from RaDR-GFP mice that carry a tandem duplication of mutant *EGFP* genes integrated at the *ROSA26* locus [20], spleen and liver data were on the same order whereas pancreas data were definitely lower in the present HPRT-dup-GFP system (Fig 2). The reason(s) for the difference is not clear but might be attributed to, for example, possible difference in the revertants’ GFP expression levels between the two systems (we have not yet obtained a positive control mouse derived from a germline mutation), or different detection methods of the mutant cells (microscopic vs. flow-cytometric). It is not understood why gene conversions do not take place in the HPRT-dup-GFP system. At least two explanations are possible: first, when a long stretch of DNA has to be replaced, legitimate homologous recombination (unequal sister chromatid exchange) is the primary choice for the cells as indicated by Aubrecht [28]. Second, the two duplicated segments are not similar enough to give rise to gene conversion. The cellular choice between homologous recombination and gene conversion might also depend on the location of the reporter construct in the genome.

In both RaDR and HPRT-dup-GFP systems, the frequency of GFP-positive revertants varied between tissues. It seems that the tissue-dependent difference is primarily due to different number of stem cells and their divisions (hierarchic structure of the tissue). It is plausible that such a difference may somehow be related to different rates of cancer incidence among tissues. In fact, a recent report indicated a highly positive correlation between lifetime cancer risk and total stem cell divisions in various human tissues [29]. Additionally, different repair systems may predominate in different types of cells. For example, homologous recombination activity is reported to be higher in mouse ES cells as compared with somatic cells [30]. Further, different levels of reporter gene expression (both the mean fluorescent intensity of the revertants and the fraction of potentially GFP-positive cells among somatic cells) between tissues may affect the observed frequencies of revertants. This possibility may well depend on the promoter used for the reporter gene, but it does not apply to RaDR mice because variations in the fraction of GFP-positive cells of *Rosa26* positive control mice varied only between 74 to 91% [20].

### Comparisons with the Dlb-1 and other systems

*Dlb-1* mutations are often screened from crypt side, and reportedly, single crypts are often occupied totally with mutant cells (e.g. [31]). In contrast, we scored revertant GFP-positive streaks in villi and rarely observed a totally green villus. The difference is likely to be due to the fact that each villus is supported by 6 to 14 crypts [23]. Therefore, even when a whole crypt is occupied with mutant stem cells, still the villus may show only a streak and is not totally green. As six or more crypts are involved to support a villus, one whole villus could be green only when stem cells in all the surrounding crypts consist of mutant stem cells. Indeed, such a rare case was observed (Fig 1K). This may happen either when a mutation occurred at an early stage of embryogenesis or after reaching adulthood followed by crypt fissions [32]. It is unfortunate that scoring GFP-positive cells on crypt (from outer surface of the small intestine) was not possible under the current experimental conditions. Observing green mutant cells on both inner and outer sides of the same intestine sample would enable us in the future to confirm the above explanations.

With regard to induced mutation frequency (Mf), Winton et al. [15] reported that unstained Dlb-1 negative ribbons appeared at a frequency of about 5×10^−4^ per villus following acute exposure of 2 Gy, and the range of induced mutation frequency was estimated as 2 to 9×10^−6^ per stem cell. Compared with these estimates, present results are notably lower; namely, means are 0.3×10^−6^ in the control and 1.6×10^−6^ in the 3 Gy groups or medians are 0 in the control and 0.5×10^−6^ in the 3 Gy group (Table 1). The difference may be due to different types of mutations detected; namely, not only forward vs. reverse mutations, but also possible contribution of loss of heterozygosity (LOH, homologous recombination between the two homologous chromosome 11’s in which the *Dlb-1* gene locates) which does not take place in the X-chromosomal HPRT-dup-GFP system. For example, mutations at an autosomal locus was often derived from LOH rather than point mutations at APRT locus in mouse fibroblasts or T cells [31] or HLA-A locus in human blood T cells [33].

Regarding the effect of deficiency of a specific gene, reported results are not concordant and confusing. For example, deficiency of p53 gene function is reported to cause large increase of recombination frequency in cultured cells bearing a tandem duplication of HSV-TK gene [34]; or tandem duplication of hygromycin gene [35] while the effect was undetected in our present system (AN unpublished observation) as reported for FYDR [36] and p^un^ systems [28]. Curiously, in the p^un^ system, no spotted mice were seen in the p53(-/-) mice while in the present study we have observed one p53(-/-) mouse in the control group that showed the extreme jackpot mutation (S1 Table). Likewise, loss of ATM gene function is reported to increase the reversion frequency by two fold in the p^un^ system [37] whereas the effect was not significant in the present system and in the primary culture of cells derived from mice bearing a direct repeat GFP construct (DR-GFP) located in *Pim1* locus [18]. In either system, individual variation of mutant cell frequencies in vivo is quite extensive and further effort is required to derive a conclusion.

### Unique problems associated with spleen cells

Clustered observations of the extreme jackpot mutations in the spleen seem to indicate that spleen cells are subjected to different kinetics from other types of somatic cells. Although direct evidence is lacking, we are inclined to believe that spleen cells are unique as to undergo antigen-stimulated clonal expansions in vivo. Indeed, our past studies on radiation-induced chromosome aberrations in blood T lymphocytes of atomic-bomb survivors often showed identical translocations among T cells of not only a restricted subset (i.e. clonal descendants of a differentiated T cell in the periphery), but also across various subsets (i.e. clonal descendants of a single stem cell) [38]. Clonal expansions of HPRT-deficient T lymphocytes in vivo are also reported in humans ([39] and references therein). Therefore, although it is practical to measure GFP-positive cells among spleen cells, especially when combined with a flow-cytometric means, caution is needed to avoid or minimize the influence of the clonal events. In this regard, it is noteworthy that natural aging might be associated with both increased number of de novo mutations and clonal expansions of mutant cells as reported in pancreas of FYDR mice [17]. In that report, mutant yellow-fluorescence-positive cells and their clonally expanded clusters were seen like nebulas in the sky, and the highest frequency observed was of the order of 10^−3^, which is comparable to the levels of extreme jackpot mutation that we observed for small intestine (Fig 3A) and liver (Fig 3C).

### Future tasks

To score the number of mutant spots in a whole organ, it is useful to introduce the compression/imaging method developed by Engelward's group [19]. However, this system may have a limited capacity to examine a large and bloody tissue like the liver; namely, GFP-positive cells located at several layers below the surface would not be easily seen. In contrast, microscopic screening of a number of frozen sections may be useful for detecting single mutant cells located deep in a tissue or for mapping the mutant cells in a tissue but is definitely time consuming and labor intensive. Our preliminary test using squashed thyroid tissue samples (S7D Fig) seems to work and may be applied to other soft tissue samples like the pancreas. Use of a flow-cytometer is another approach provided that the expression time for mutated cells to fully express the mutant phenotype can be set properly (but this may be tissue dependent) so that the effect of stem-cell-derived clonal expansion may be minimized.

The present HPRT-dup-GFP system seems to have a potential use for screening environmental chemicals. Since many tissues can be screened at a time on the same animals, it may contribute to saving the number of animals required. For this purpose, however, reductions of both the background frequency and inter-individual variation are essential. One possibility is to administer 6-thioguanine to the animals during fetal life and early postnatal development so that preexisting stem cell-derived GFP-positive descendants may be largely eliminated prior to the administration of test chemicals. Another approach is to introduce a cell-specific gene expression system that can be triggered by the GFP proteins as proposed by Tang et al [40]; for example, conditional expression of a caspase or a toxin gene in GFP-positive cells may eliminate preexisting mutant cells. In either case, a large fraction of HPRT- or GFP-positive cells needs to be effectively targeted and eliminated.

Last, the implications of the extreme jackpot mutations observed in solid tissues should be considered. Because individual somatic cells are derived from stem cells in vivo, they are under hierarchical structures and not independent entities. Therefore, when we think of somatic mutations in vivo, development of an individual can be compared to a fluctuation tests in vitro that start with a single cell per culture (but without differentiation). When a mutation occurs early, all the descendants are mutants and hence mutant frequency is enormously high while the probability of a spontaneous mutation to occur among a small number of cells is quite low. By contrast, when a mutation occurs late, the mutant frequency is not much affected while the probability of a mutation to arise in a culture is much elevated.

In this regard, past cytogenetic studies revealed that aneuploid cells are commonly observed in blood lymphocytes of normal individuals, and the frequency is quite low when young but increases to a few percent among the elderly [41]. Recent development of FISH technique also made it possible to estimate the frequency of aneupoid cells among non-dividing cells. A substantial number of reports indicated that aneuploid cells are surprisingly common; for example, 10 to 30% in brain cells [42–44]. Because blood lymphocytes are one of the most vigorously dividing cells in vivo, they are sensitive to negative selection if the doubling time is increased in aneuploid cells, which is indeed the case in yeast [45]. In contrast, aneuploid or mutant cells comprising a solid tissue may be less sensitive to the pressure of negative selection because cell turnover is much slower than in hemato-lymphoid cells once the tissues were established. The aneuploidy cells might contribute to the development of adult onset diseases [46],[47]. In a more recent study using single cell sequencing technique, however, aneuploid levels across different tissues including brain cells were much lower than previously reported, raising a need to confirm the past observations with a new methodology [48].

The observed GFP-positive cells as clusters in partial villi, not crypts (Fig 3B), may look unusual because reversion mutations may appear to have occurred in a spotted way in the small intestine. Here, let us recall that a whole villus cannot be green unless the supporting 4 to 16 crypts were all green, and a whole crypt cannot be green unless all stem cells were green. Clusters of partial villi similar to Fig 3 were observed among chimeric mice produced by aggregation of two early embryos with distinguishable genotypes (*Dlb-1* ^*a*^ vs. *Dlb-1* ^*b*^ cells) [49]. The authors suggested an intermingling nature of intestinal stem cells, which seems to be similar to our conditions of mice with extreme jackpot mutations.

In the present study, we found two mice out of 278 of which at least one solid tissue showed extreme jackpot mutations. This means that if we imagine mutant cell frequencies at any one of many genes, say, 100 genes related to DNA repair or cancer development, then any individual mouse is likely to carry one tissue or two that contains a large number of cells mutated at such a locus. While in vitro baseline frequencies of GFP-positive mutant cells in embryonic fibroblasts derived from the HPRT-GFP mice is not largely different from that of the forward mutations at the HPRT locus (order of 10^−6^ to 10^−5^; AN unpublished observation), further studies are required to know if the present findings may be expanded to ordinary forward mutations of many genes in the genome or may be restricted to genes containing large blocks of homology sequences. Toward this goal, it would be desired to create a mouse model which detects forward mutations in somatic cells in situ because repeat sequences may be inherently unstable [50]. We had previously created such a system in cultured human cells using the Tet O-GFP/TetR system [51]. Such a new system, if successfully introduced in the mouse, would provide hitherto unrecognized information on cell-based body structure and aid better understanding of cancer mechanisms and its ultimate prevention.

## Conclusion

We have successfully created a mouse system comprising a partial duplication of endogenous *HPRT* gene conjugated with *GFP* gene at the last exon (HPRT-dup-GFP system). Such mice were found to bear GFP-positive cells in many tissues thus far examined while the frequency of GFP-positive cells varied not only among individuals but also among tissues investigated. Mutagenic effect of ionizing radiation (3 Gy) was only modest (about two-fold increase) primarily due to the presence of large individual variations. Another notable observation was that two mice out of about 300 had too many GFP-positive cells to score in small intestine or liver. The results indicate that we are likely to be composed of so many mutant cells, much more than we can imagine, and some of them might be related to the development of adult onset diseases. By the aid of new biologic technologies, we have just started to learn about the complex nature of somatic mutations occurring in vivo associated with individual development and aging. Further studies will certainly add much more information in this interesting research field.

## Supporting Information

S1 File Supplementary Materials and Methods. (DOCX)

S1 Table The number of mice which showed extreme jackpot mutations in at least one tissue examined among the total number of mice bearing the same genotype or conditions. (DOC)

S1 Fig Outlines for generating recombinant ES cells bearing an X-chromosome with CAG-HPRT-dup-GFP sequences. (TIF)

S2 Fig Outline for creation of the first knock-in vector (upper) and second knock-in vector (lower). For the details, see Supplementary Materials and Methods.
(TIF)

S3 Fig Confirmation of the structure of HPRT-dup-GFP allele and characterization of revertant alleles by Southern analysis. Expected and observed sizes of Southern bands of wild-type (WT), knocked-in (KI), or revertant (REV) HPRT allele are shown. Note that 2.2kb band is observed only in KI allele. Positions of PCR primers and the size of PCR products are shown in the upper panel.
(TIF)

S4 Fig Characterization of different ES clones bearing the knock-in allele and their revertant clones. Locations of PCR primers and the expected sizes of PCR products are indicated in the upper panel. Lower left; Four clones bearing knock-in allele were examined for the presence of 7.8, 2.5, and 0.45kb bands. Lower right; Total 10 revertant clones were examined for the presence of 8.6kb band that is only possible when one of the two duplicated segment containing the neo marker was lost. 7.8kb band corresponds to KI allele, 8.6kb band revertant allele. Bands that appear above these bands are technical artefact.
(TIF)

S5 Fig Characteristics of revertant cells. **A)** Flow-cytometric measurement of parental CAG-HPRT-dup-GFP ES cells and their revertants (mutants). **B)** Two revertant colonies observed under a fluorescent microscopy.
(TIF)

S6 Fig Detection and measurement of HPRT-GFP mRNA by real-time quantitative RT-PCR. **(A)** A schematic view of homologous recombination-mediated reversion and production of HPRT-GFP fusion transcript. **(B)** Amplified transcripts. Mouse XPA gene transcript was used as an internal control. **(C)** Amplification profile which indicates that the mouse tail tissue contained revertant GFP-positive cells at a frequency of one in about 10^5^ cells assuming that ES cells and mouse somatic cells in a tail tip contain a similar level of mRNA. Note that the curves of ES HPRT-GFP and 1–35 mouse HPRT-GFP show a difference of nearly 10 cycles (which corresponds to about 1,000 times difference) in the amount of cDNA while the cDNA sample from ES HPRT-GFP cells (revertants) were diluted by 100 times before being subjected to the PCR.
(TIF)

S7 Fig Possible mechanisms of reversion mutations of HPRT-dup-GFP allele to give rise to HPRT-GFP fusion proteins. **A)** Simple recombination caused by an unequal sister chromatid exchange (a red cross) or an intra-molecule exchange (dotted lines). Note that the resulting GFP-positive cells are devoid of the neo marker, which is what was observed. **B)** A possible model that involves a single strand invasion at a replication folk and replaces truncated exon 8 with a distantly located normal exon 8 along with exon 9 and GFP gene in the second duplication. Single strand invasion may start at anywhere between intron 5 and exon 8 of the first duplication but ends after the GFP gene. If the 3’ end of the invaded DNA returned to the starting point of the invasion, the event may be called a homology-mediated insertion event (red arrow). Note that in this scenario, neo marker is retained while two GFP genes remain (one is actively transcribed while the other is not). It is noted that this scenario is not likely because the revertant GFP+ cells had always lost the neo marker that owns its own promoter to express (MC promoter-neo) and does not require the HPRT promoter. On the other hand, if the 3’ end of the invaded DNA returned to its homology sequences (red broken arrow), it is a double recombination and is indistinguishable from unequal sister chromatid exchange process shown in **A)**.
(TIF)

S8 Fig GFP-positive cells in a variety of tissues (indicated as white arrows). (TIF)
