# Supplementary material for: Creation of Mice Bearing a Partial Duplication of HPRT Gene Marked with a GFP Gene and Detection of Revertant Cells In Situ as GFP-Positive Somatic Cells
Source: PLoS One. 2015 Aug 21;10(8):e0136041. doi: 10.1371/journal.pone.0136041 (PMC4546575; doi:10.1371/journal.pone.0136041)
Supplement: S1 File — (DOCX) [file pone.0136041.s001.docx]

**Supplementary Materials and Methods**

**Creation of ES cells bearing HPRT-dup-GFP sequences**

Partial duplication of endogenous HPRT gene was created by using a knock-in vector which carries genomic DNA fragments consisting of *HPRT* gene intron 5 to exon 8 (7.8Kb) and intron 5 to exon 9 sequences (8.6Kb) (S1Fig.). At the 3’ end of the first fragment was inserted a floxed neo gene cassette (at the truncated exon 8), and at the 3’ end of the second fragment was inserted a GFP ORF in frame so that the stop codon of the *HPRT* gene is removed (HPRT-dup-GFP vector); S2 Fig. However, recombinant mice derived from the ES cells did not show any GFP-positive mutant cells in vivo. To enhance expression levels of the endogenous *HPRT* gene, second gene targeting was performed in the HPRT-dup-GFP ES cells to replace the endogenous mouse *HPRT* promoter with a CAG promoter flanked by a FRT-puro cassette (S2 Fig.).

**Reversion mechanisms**

Deletion of one copy of the duplicated segments results in conversion of the phenotype from HPRT-deficient (6TG-resistant) and GFP-negative to HPRT-proficient (6TG-sensitive and HAT-resistant) and GFP-positive.

**Flow cytometric results of parental and revertant ES cells**

Revertant ES cells (green mutants) showed fluorescence intensities >200-fold higher than the parental cells (S5A Fig.), and the colonies are fully green (S5B Fig.).

**Construction of plasmid vectors and their knock-ins into ESR1 cells (S2 Fig.)**

1) First knock-in: Mouse genomic DNA fragments derived from ESR1 cells were isolated by LA-PCR (Takara-Bio, Japan) and the sequences were verified. First, a 8.6 Kb of HPRT genomic DNA was PCR amplified, which includes intron 5 to exon 8 and a part of exon 9 where stop codon is deleted (it corresponds to #120440 to #128993 of mouse BAC clone BX649621). And the product was cloned into pCR2.1TOPO (Invitrogen) to make pCR-mhprt8.6. The 8.6 Kb of Eco RI fragment was then inserted into *Eco*RI site of a GFP vector, pQBI25-fNI (Wako, Japan). The resulting plasmid, pQBI-mhprt8.6, carries a GFP ORF in-frame of the HPRT last codon, which implies that HPRT-GFP fusion protein will be produced if the HPRT gene structure is complete. By using the pQBI-mhprt8.6 as template, (1) *Sal*I sequence-tagged LA-PCR was performed to amplify 9.5 Kb of HPRT-GFP sequences, and the resulting amplicon was cloned into a pTA vector (Toyobo, Japan) to make pTA-8.6GFP. The primer sequences were GTCGACATGTGTTTTGCTGCTCATGA, and GTCGACTATTGTCTTCCCAATCCTC. After the verification of the sequences, the plasmid was digested with *Sal*I, and 9.5 Kb fragment was isolated. (2) *Bam*HI sequence-tagged LA-PCR was performed to amplify 7.8 Kb of *HPRT* which included intron 5 to a part of exon 8. The primer sequences were GGATCCATGTGTTTTGCTGCTCATGA, and GGATCCCAAATCCCTGAAGTACTCAT. Then the 7.8 Kb fragment was cloned into a pTA to make plasmid pTA-left. After the verification of the sequences, the 7.8 Kb fragment was isolated by *Bam*HI digestion. A Neo resistance gene unit was isolated from pMC1-neo-PA (Stratagene) by *Xho*I and *Bam*HI digestion and inserted into *Eco*RV site of a pBS-loxP vector by blunt end ligation. The resulting plasmid, pBS-neo-loxP was digested with *Bam*HI, and the 7.8 Kb of left arm DNA was inserted to make pBS-neo-loxP-7.8. Then it was digested with *Sal*I and the above-mentioned 9.5 kb *Sal*I fragment (8.6-GFP) was combined to make pBS-neo-loxP-7.8-8.6-GFP. Next, downstream sequences of mouse *HPRT* gene was isolated as an Apa I sequence-tagged 2.3 Kb fragment by LA-PCR. The primer sequences were GGGCCCTTTGGGACCAAAAGTCCTGT, and GGGCCCCTGGGAATTGAACTCAGGAC. It locates in downstream of *HPRT* stop codon, corresponding to #129991 to 132260 of the BAC. Then the 2.3 Kb fragment was inserted into pTA to make pTA2.3. The 2.3 Kb *Apa*I fragment was then isolated and inserted into *Apa*I site of pBS-neo-loxP-7.8-8.6-GFP to make pBS-neo-loxP-7.8-8.6-GFP-2.3. A MC-DTA cassette was isolated by *Kpn*I digestion of pMC-DTA1, and inserted into *Kpn*I site of the pBS-neo-loxP-7.8-8.6-GFP-2.3. The resulting plasmid, pBS-neo-loxP-7.8-8.6-GFP-2.3-DTA was digested with *Bss*HII, and 22 Kb fragment containing *HPRT*-partial duplicate-GFP-DTA was used for ES cell targeting.

2) Second knock-in (S2Fig.): BAC library from mouse129Sv was purchased from CHORI (BAC PAC resource center, CA) and screened, and the clones containing mouse *HPRT*, RP22-98-E9 and RP22-216F2, were isolated. From these plasmids, DNA fragments for the left and the right arm of targeting vector were isolated and inserted into pBS-SK plasmid: Left arm consists of 11.4 Kb of *Nhe*I fragment, which is located at 3.8 Kb upstream of mouse HPRT ATG and it corresponds to base sequences of #80888 to #92288 of above mentioned BAC BX649621 sequences. Right arm consists of 1.4 Kb of a *Bbv*C1 fragment, which also corresponds to the sequences from #17212 to #18653, in which HPRT ATG is located at #17326. pBS-DTA1 [**51**] was digested with *Ban*III and after blunting by fill-in reaction, the 1.4 Kb of *Bbv*C1 fragment which was also blunted, was combined to make pBS-BbvC1-1.4DTA. A 2.8 Kb of 1.4-DTA fragment was isolated by *Hind*III and *Xho*I digestion. After the blunting of both ends, it was inserted into blunt-ended *Xho*I site of ploxP-neo-CAG plasmid. The resulting plasmid, ploxP-neo-CAG-1.4-DTA, was digested with *Sal*I and *Kpn*I, and 4.1 Kb of CAG-1.4-DTA fragment was isolated and blunted. Plasmid pBS-FRT-pgk-puro was digested with *Apa*I and blunted, and then the 4.1 Kb fragment was inserted to make pPGK-puro-CAG-1.4-DTA. The plasmid was digested with *Not*I and blunt-ended. A 11.4 Kb *Nhe*I fragment was cloned into PBS-SK, and then the 11.4 Kb fragment was isolated by *Not*I and *Eco*RV digestion. Following the blunting of both ends, it was inserted into the *Not*I site (blunt) of pPGK-puro-CAG-1.4-DTA, to make it finally as pPGK-puro-Target. 3) The target vector was digested with *Swa*I, and used for targeting the HPRT-dup-GFP-ESR1 cells (second time targeting). With these procedures, a partial duplication of HPRT gene bearing GFP sequences, and replacement of endogenous promoter were completed. The recombinant ES cells became resistant to 6TG, G418, and puromycin (S1 Fig.). Knock-in allele was confirmed by Southern and PCR analyses (S3 and S4 Figs.). All of the revertant clones exhibited resistance to HAT, puromycin, and sensitive to G418, either spontaneous or radiation-induced, were found to bear a deletion of one of the duplicated segments. In this system, false-positive cells are unlikely to emerge.

After the germline transmission of the ES cells, the knock-in mice were repeatedly mated with C57BL/6J to make stable genetic background.
