# Supplementary material for: Creation of Mice Bearing a Partial Duplication of HPRT Gene Marked with a GFP Gene and Detection of Revertant Cells In Situ as GFP-Positive Somatic Cells
Source: PLoS One. 2015 Aug 21;10(8):e0136041. doi: 10.1371/journal.pone.0136041 (PMC4546575; doi:10.1371/journal.pone.0136041)
Supplement: S1 Table — (DOC) [file pone.0136041.s002.doc]

**S1 Table** The number of mice which showed extreme jackpot mutations in at least one tissue examined among the total number of mice bearing the same genotype or conditions.

| Mouse genotype and conditions | Control | 3 Gy exposure |
| --- | --- | --- |
| P53(+/−) | 0 / 8 | 1^1)^ / 14 |
| P53(−/−) | 1^2)^ / 7 | 0 / 9 |
| ATM(+/−) | 0 / 4 | 0 / 3 |
| ATM(−/−) | 0 / 3 | 0 / 4 |
| Wild-type F1 derived from irradiated spermatogonia |  | 2^3),4)^ / 80 |
| Wild-type F1 derived from irradiated spermatozoa |  | 2^5),6)^ / 28 |
| P53(+/−) F1 derived from irradiated p53(−/−) spermatogonia |  | 1^7)^ / 15 |
| Subtotal | 7 / 175 | |
| Wild type | 0 / 56 | 0 / 47 |
| Total | 7 / 278 | |

^1)^ Mouse ID #445 in Table 2, ^2)^#413, ^3)^#144, ^4)^#484, ^5)^#22, ^6)^ #26, ^7)^#47
