# Supplementary figures and images for: Creation of Mice Bearing a Partial Duplication of HPRT Gene Marked with a GFP Gene and Detection of Revertant Cells In Situ as GFP-Positive Somatic Cells
Source: PLoS One. 2015 Aug 21;10(8):e0136041. doi: 10.1371/journal.pone.0136041 (PMC4546575; doi:10.1371/journal.pone.0136041)

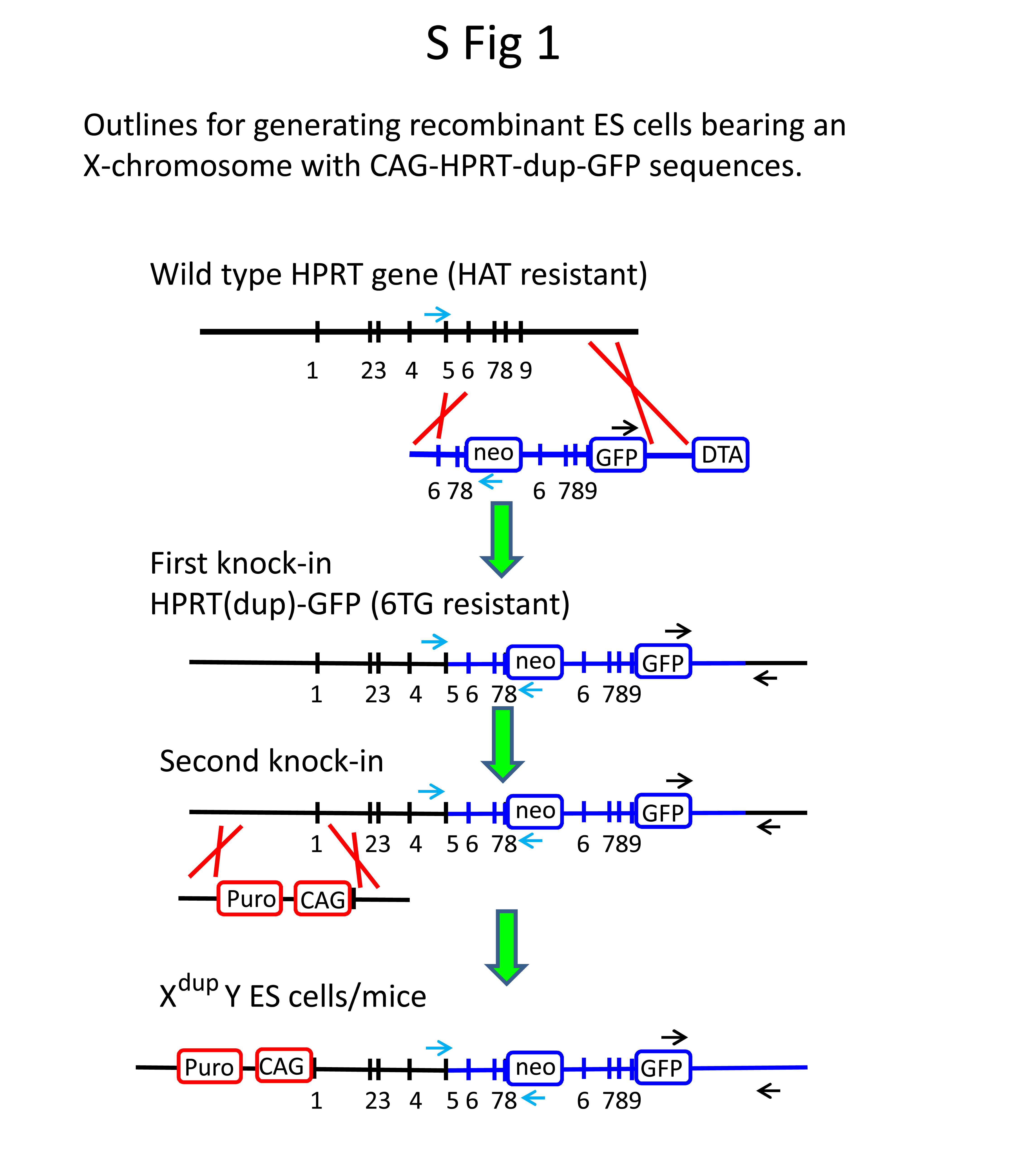

Supplement: S1 Fig — (TIF) [file pone.0136041.s003.tif]

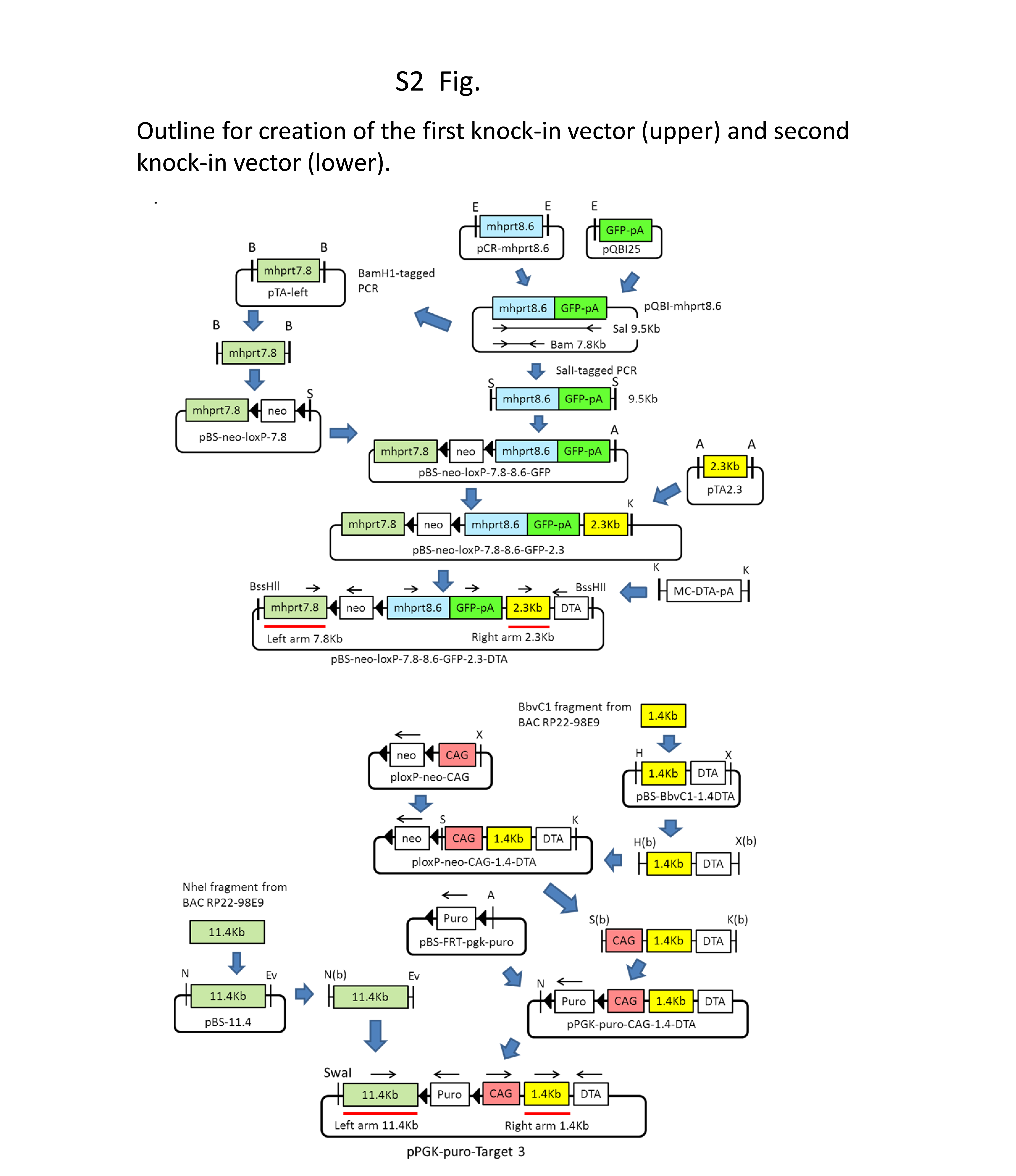

Supplement: S2 Fig — For the details, see Supplementary Materials and Methods. (TIF) [file pone.0136041.s004.tif]

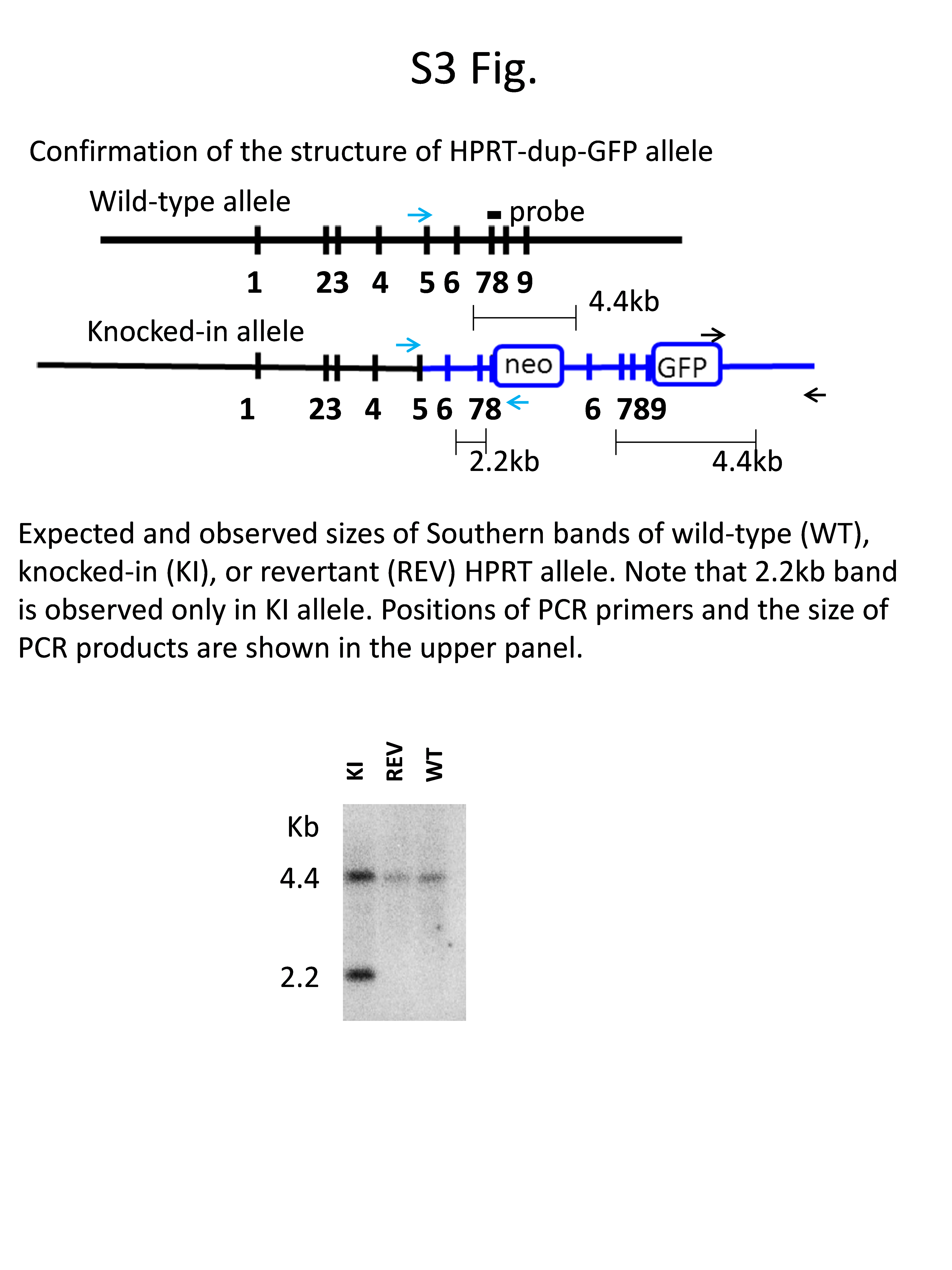

Supplement: S3 Fig — Expected and observed sizes of Southern bands of wild-type (WT), knocked-in (KI), or revertant (REV) HPRT allele are shown. Note that 2.2kb band is observed only in KI allele. Positions of PCR primers and the size of PCR products are shown in the upper panel. (TIF) [file pone.0136041.s005.tif]

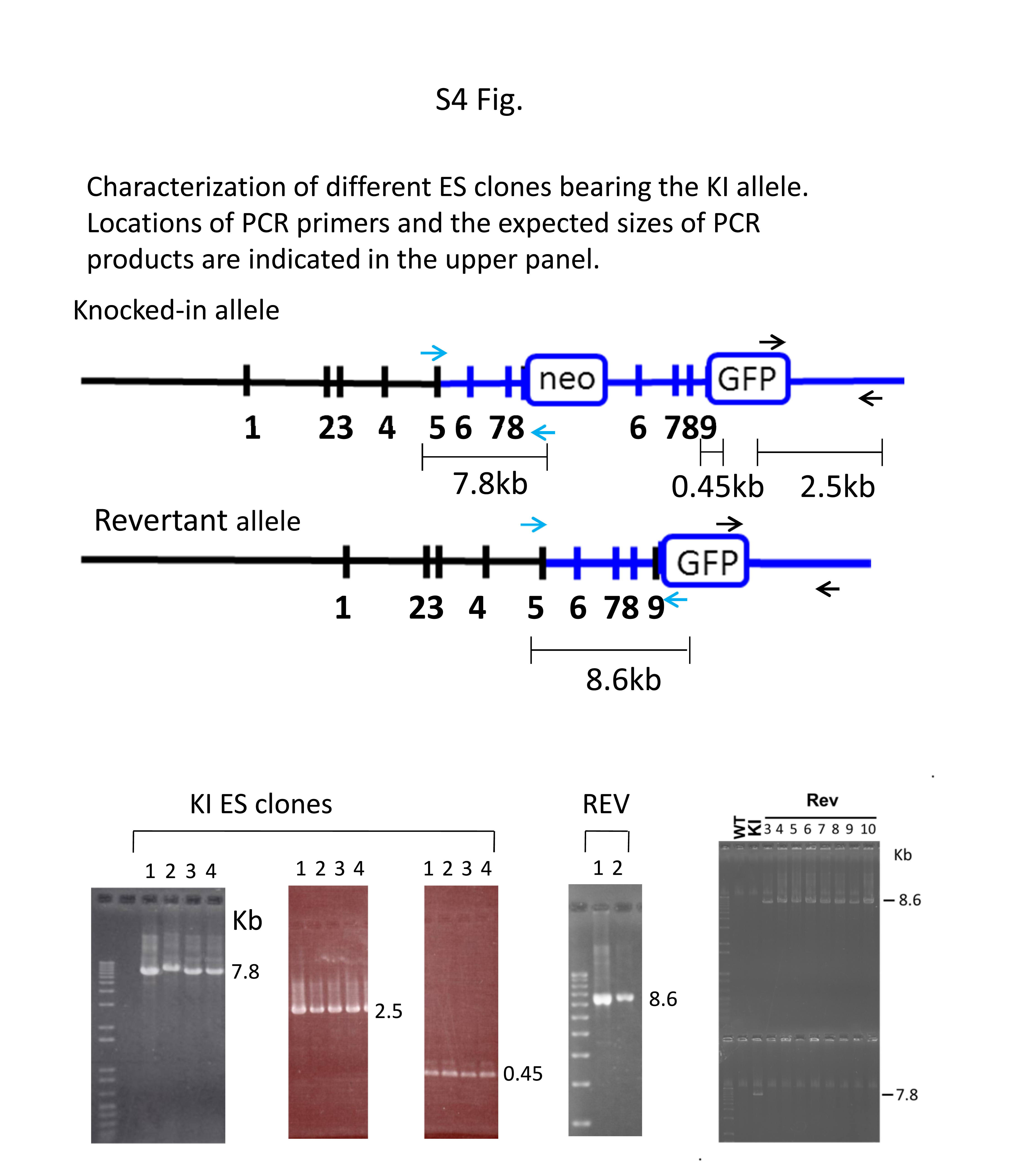

Supplement: S4 Fig — Locations of PCR primers and the expected sizes of PCR products are indicated in the upper panel. Lower left; Four clones bearing knock-in allele were examined for the presence of 7.8, 2.5, and 0.45kb bands. Lower right; Total 10 revertant clones were examined for the presence of 8.6kb band that is only possible when one of the two duplicated segment containing the neo marker was lost. 7.8kb band corresponds to KI allele, 8.6kb band revertant allele. Bands that appear above these bands are technical artefact. (TIF) [file pone.0136041.s006.tif]

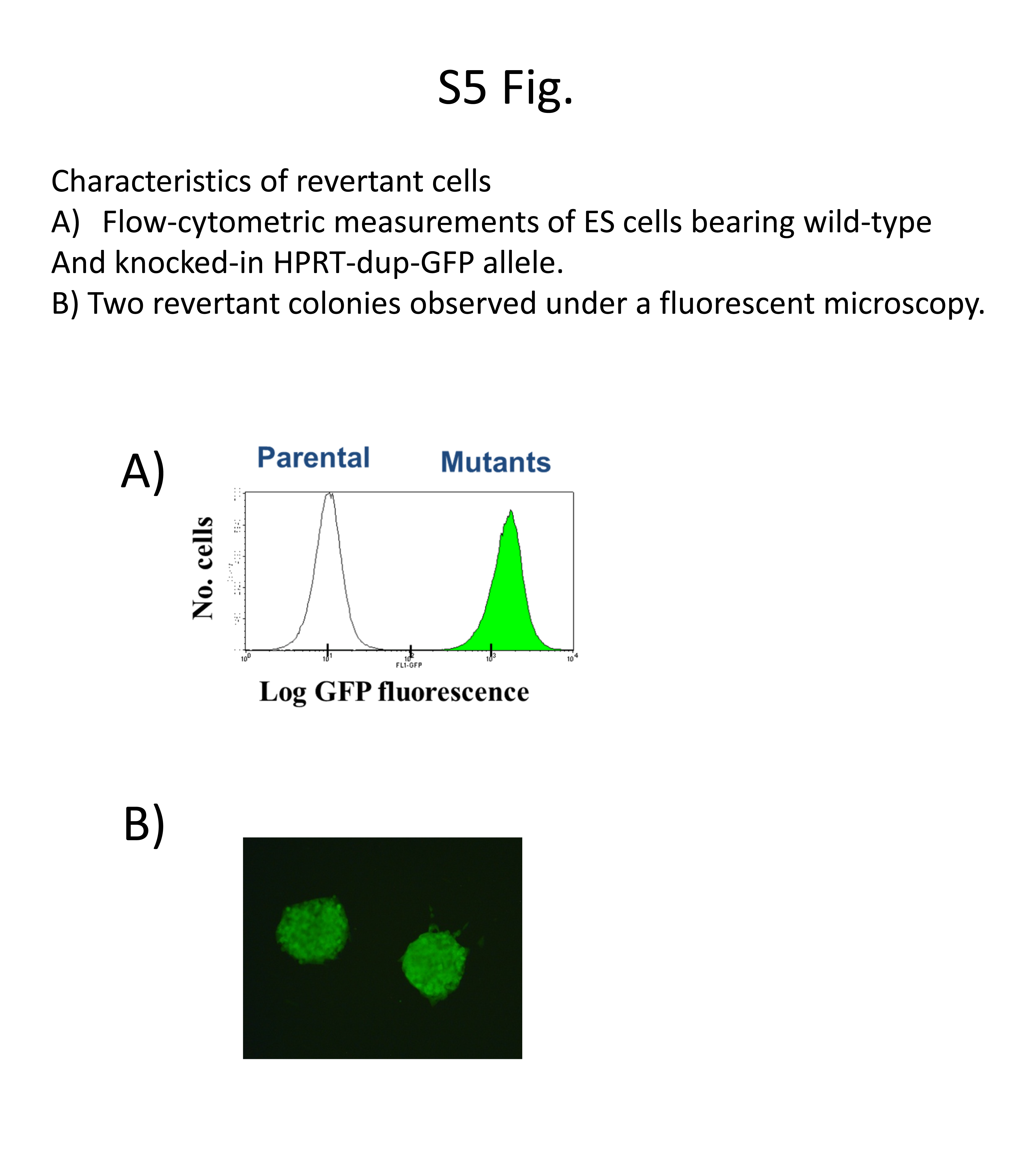

Supplement: S5 Fig — A) Flow-cytometric measurement of parental CAG-HPRT-dup-GFP ES cells and their revertants (mutants). B) Two revertant colonies observed under a fluorescent microscopy. (TIF) [file pone.0136041.s007.tif]

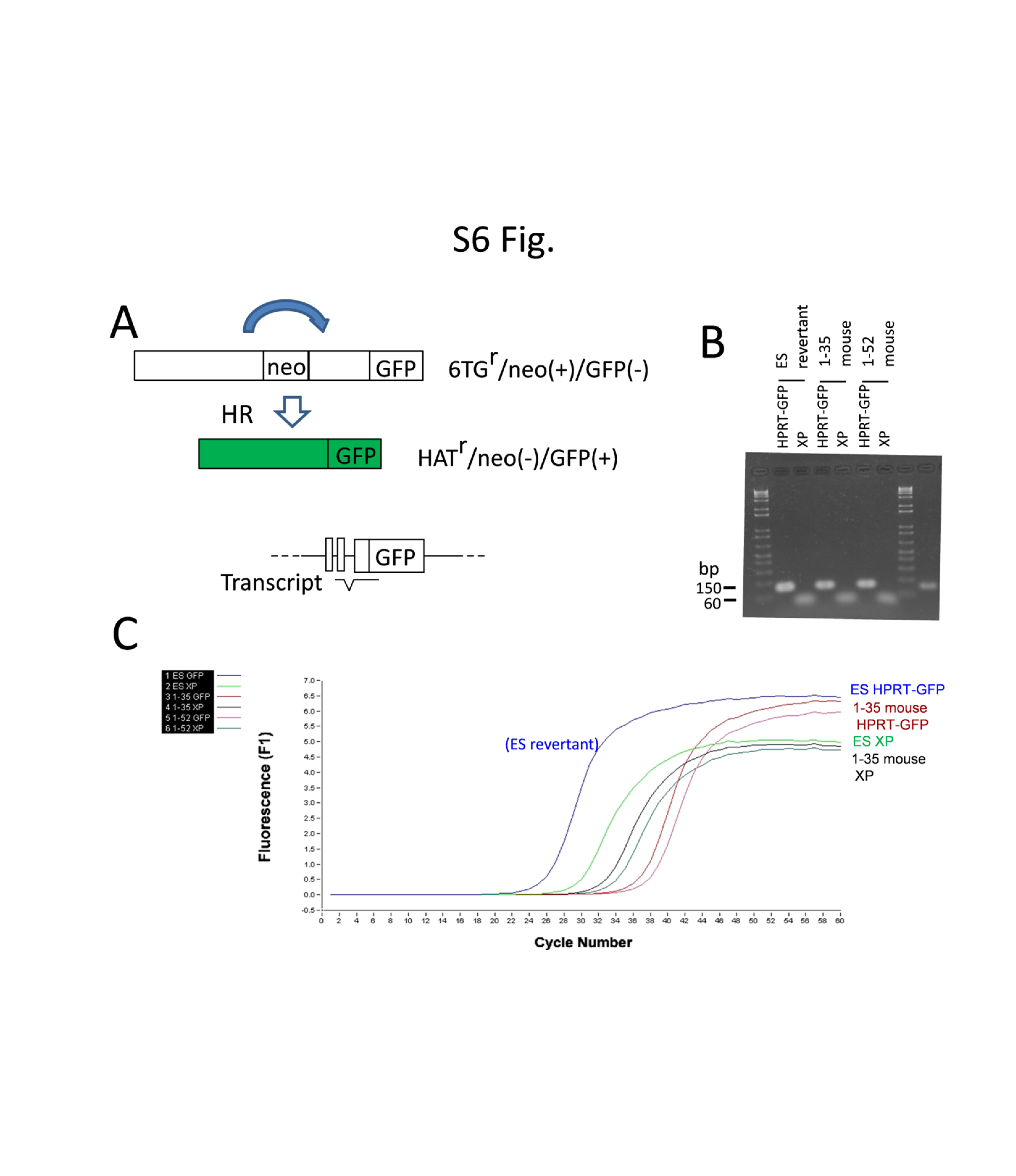

Supplement: S6 Fig — (A) A schematic view of homologous recombination-mediated reversion and production of HPRT-GFP fusion transcript. (B) Amplified transcripts. Mouse XPA gene transcript was used as an internal control. (C) Amplification profile which indicates that the mouse tail tissue contained revertant GFP-positive cells at a frequency of one in about 105 cells assuming that ES cells and mouse somatic cells in a tail tip contain a similar level of mRNA. Note that the curves of ES HPRT-GFP and 1–35 mouse HPRT-GFP show a difference of nearly 10 cycles (which corresponds to about 1,000 times difference) in the amount of cDNA while the cDNA sample from ES HPRT-GFP cells (revertants) were diluted by 100 times before being subjected to the PCR. (TIF) [file pone.0136041.s008.tif]

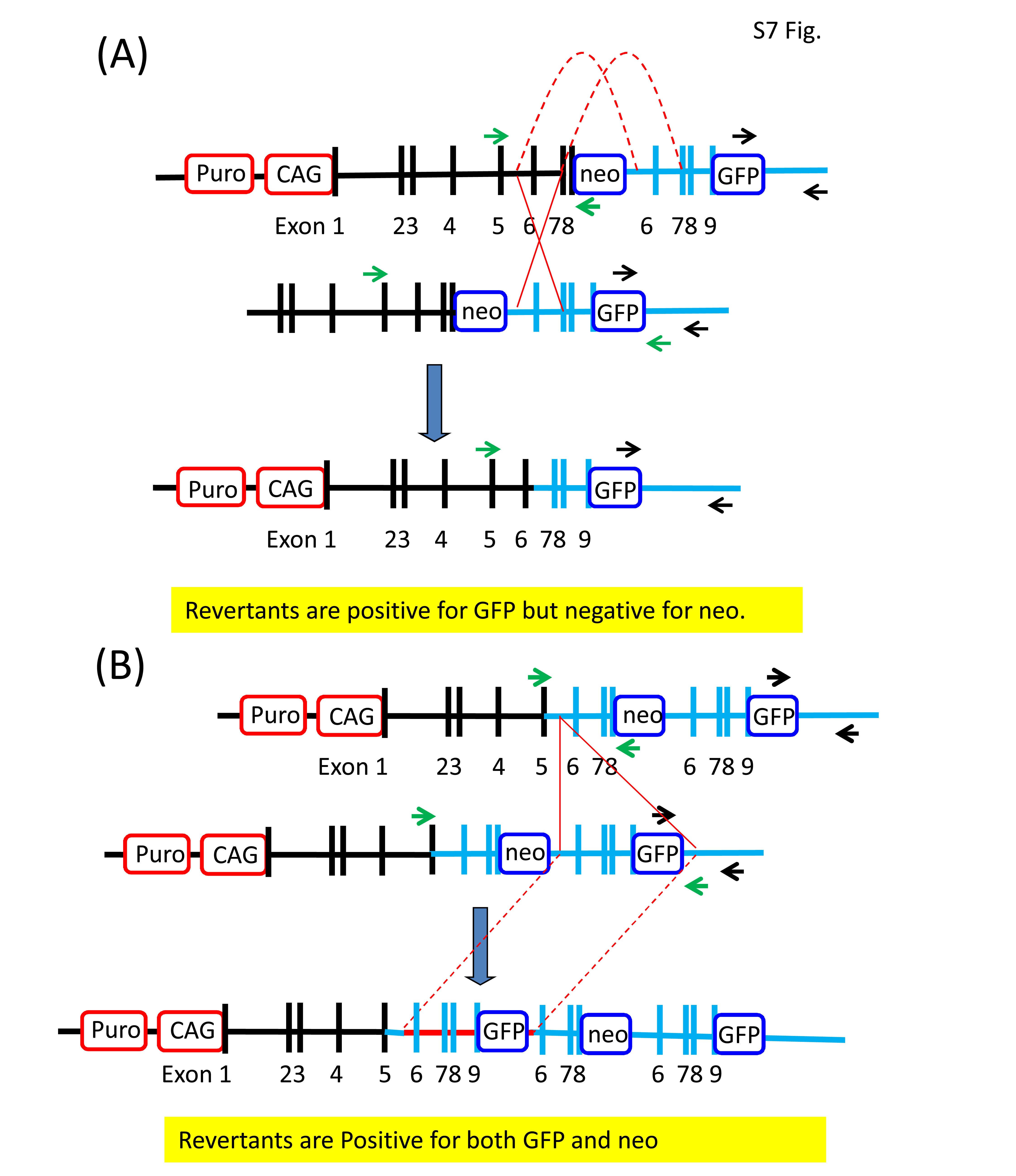

Supplement: S7 Fig — A) Simple recombination caused by an unequal sister chromatid exchange (a red cross) or an intra-molecule exchange (dotted lines). Note that the resulting GFP-positive cells are devoid of the neo marker, which is what was observed. B) A possible model that involves a single strand invasion at a replication folk and replaces truncated exon 8 with a distantly located normal exon 8 along with exon 9 and GFP gene in the second duplication. Single strand invasion may start at anywhere between intron 5 and exon 8 of the first duplication but ends after the GFP gene. If the 3’ end of the invaded DNA returned to the starting point of the invasion, the event may be called a homology-mediated insertion event (red arrow). Note that in this scenario, neo marker is retained while two GFP genes remain (one is actively transcribed while the other is not). It is noted that this scenario is not likely because the revertant GFP+ cells had always lost the neo marker that owns its own promoter to express (MC promoter-neo) and does not require the HPRT promoter. On the other hand, if the 3’ end of the invaded DNA returned to its homology sequences (red broken arrow), it is a double recombination and is indistinguishable from unequal sister chromatid exchange process shown in A). (TIF) [file pone.0136041.s009.tif]

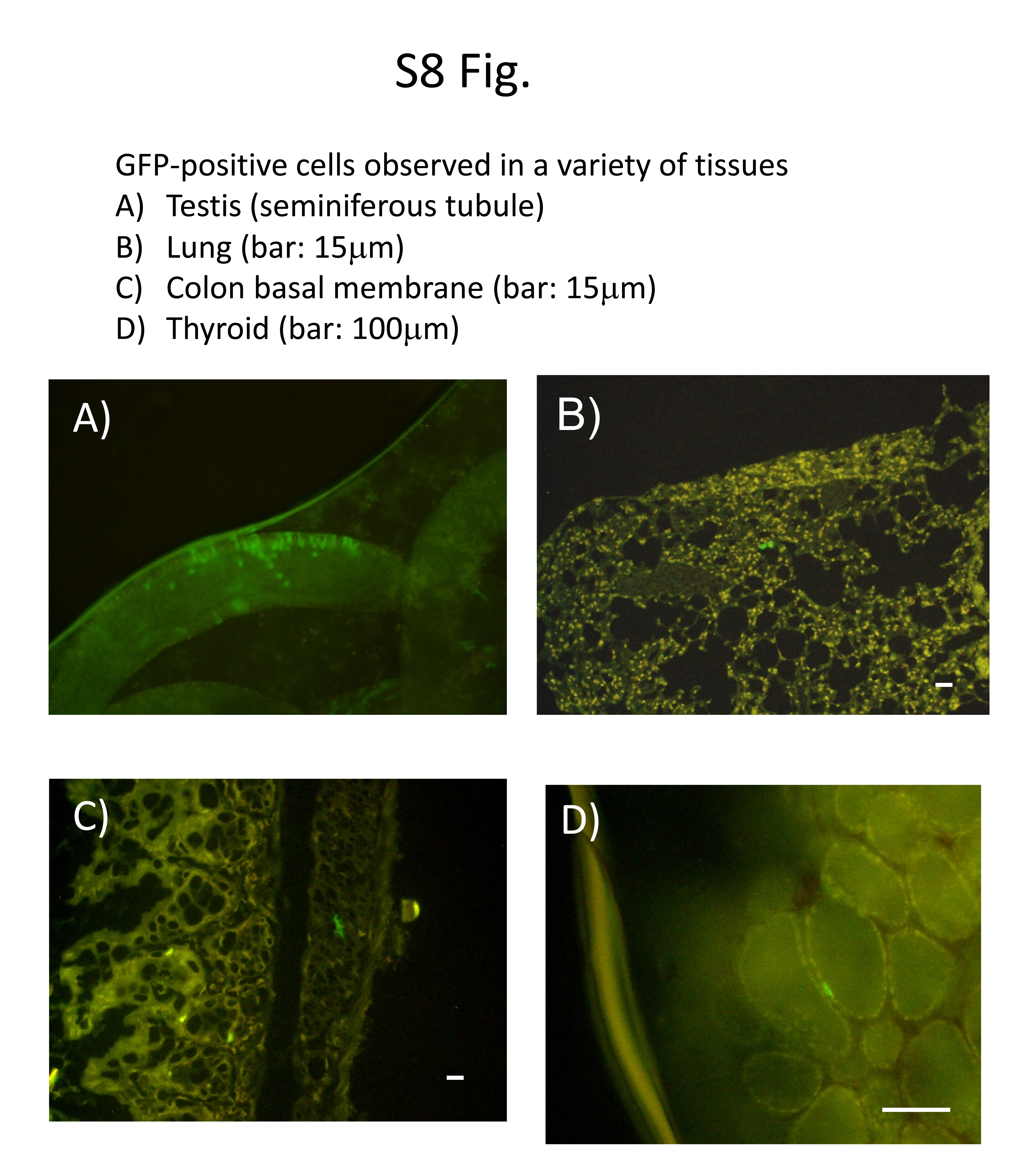

Supplement: S8 Fig — (TIF) [file pone.0136041.s010.tif]
